# Deficiency of migration inhibitory factor influences the gut microbiota of C57BL/6 mice infected with *Plasmodium berghei* ANKA

**DOI:** 10.3389/fmicb.2022.978644

**Published:** 2022-08-12

**Authors:** Yiting Xie, Wei Guan, Yanqing Zhao, Siyi Yan, Kun Guo, Sirui Chen, Xinyi Hu, Haimei Shi, Jian Li

**Affiliations:** Department of Human Parasitology, School of Basic Medicine, Hubei University of Medicine, Shiyan, China

**Keywords:** *Plasmodium berghei*, macrophage migration inhibitory factor, cerebral malaria, gut microbiota, 16S rRNA

## Abstract

Cerebral malaria (CM), as one of the most common complications in severe malaria, has threatened millions of individuals’ neurological health and even their lives. Macrophage migration inhibitory factor (MIF), a pleiotropic proinflammatory factor in humans, seems to be a risk factor for death in patients with CM, but its functional mechanism remains unclear. To verify whether affecting the intestinal microbes of the host was one of the mechanisms by which MIF regulates CM, C57BL/6 mice, including WT + PbA, MIF-KO + PbA and their uninfected controls, were sent for 16S rRNA-based sequencing targeting the V4 region of the intestinal microbiota through the Illumina MiSeq platform. The results showed that OTU clustering, alpha and beta diversity in the four groups involved had evident variation. The relative abundance at different taxonomic levels, especially the dominant intestinal flora, was obviously changed. The LEfSe analysis screened out several biomarkers, including significantly reduced *Ligilactobacillus* (*Lactobacillus murinus*) in WPbA mice compared to the WT group and *Akkermansia* (*Akkermansia_muciniphila*) in KPbA mice compared to the WPbA group. For MIF KO groups, mice infected with PbA or uninfected showed significant enrichment of producers of short-chain fatty acids, including *Dubosiella* and *Faecalibaculum* (*Faecalibaculum rodentium*) in KPbA, and *Lachnospiraceae_NK4A136_group* and *Firmicutes_bacterium_M10-2* in KO. This study not only further proved the gut microbiota changes in C57BL/6 mice caused by PbA infection, but also found that MIF deletion directly affected the changes in the gut microbiota of C57BL/6 mice before and after PbA infection. This finding reveals a potential mechanism by which MIF regulates CM. Combining MIF with potential microbial biomarkers will provide a promising idea to develop combined drugs for improving CM in the future.

## Introduction

Malaria caused by *Plasmodium* infection is a widespread infectious disease in tropical and subtropical regions. According to the latest report by the World Health Organization (WHO; WHO, 2021), the estimated malaria cases reached up to 241 million worldwide in 2020 and were distributed in 85 malaria-endemic areas. Among these, approximately 627,000 deaths occurred. Most of them (95%) appeared in Africa, followed by Southeast Asia (2%), and 77% of patients were children under 5 years of age. Severe malaria is multisyndromic. Its most common manifestations are severe malaria anemia (SMA) and cerebral malaria (CM). Without timely and effective treatment, the mortality rate is fairly high (WHO, 2021). Of these, CM is a serious neurological complication in malaria patients and is mainly caused by infection with *Plasmodium falciparum*. Even with effective antimalarial therapy and intensive care, CM survivors may also develop long-term neurological deficits (Carter et al., 2005; Boivin et al., 2007; John et al., 2008). Therefore, actively improving the disease process of CM is a beneficial attempt to improve the survival rate and quality of life of patients, especially in high-endemic areas of *P. falciparum*.

Several studies have shown that human macrophage migration inhibitory factor (MIF), a pleiotropic proinflammatory factor, is closely related to the occurrence and development of various parasitic diseases (Rosado Jde and Rodriguez-Sosa, 2011; Bozza et al., 2012). Studies have found that MIF may be implicated in the pathogenesis of CM (Clark and Awburn, 2002; Clark et al., 2003; Jain et al., 2009). Of these, a study of clinical cases suggested that elevated MIF levels seemed to be a risk factor for death in patients with CM (Jain et al., 2009). These results suggested that MIF may be a potential molecular target for improving CM and even other severe malaria. Conversely, another study found that MIF had a protective role in CM (Awandare et al., 2007), implying that MIF probably has both protective and adverse effects against malaria. However, as a key rationale for the development of MIF-targeted therapeutic regimens, the role and mechanism of MIF in the regulation of malaria, especially CM, remain unclear. *In vivo*, MIF is abundantly expressed in gastric, intestinal, and colonic epithelia, as well as in the epithelium of human intestinal xenografts (Maaser et al., 2002). Additionally, MIF is involved in the maintenance of physiological microbiota diversity and immune surveillance, thereby enabling normal intestinal barrier functions (Vujicic et al., 2018). The absence of MIF increased the intestinal permeability (Vujičić et al., 2020). These results all indicated the important role of MIF in maintaining host intestinal function. Several studies have shown that malaria may lead to host intestinal lesions (Taniguchi et al., 2015; Shimada et al., 2019). Especially in patients infected with *P. falciparum*, frequent gastrointestinal symptoms, such as abdominal pain and diarrhea, and changes in the composition of the gut microbiome were observed (Taniguchi et al., 2015). Therefore, a speculation was boldly proposed that perhaps affecting the patient’s intestinal function, such as changing the microbial community of the host intestine, acts as one of the mechanisms by which MIF regulates CM.

Another important reason that the study mainly focused on the regulation of MIF on gut microbiome of CM individuals is as follows. In recent years, regulating the development of diseases by artificially interfering with gut microbes (e.g., adding probiotic yogurt to the daily diet) seems to be an emerging and promising strategy for the prevention and treatment of various diseases (Kataoka, 2016; Clemente et al., 2018; Grochowska et al., 2018). A study by Yilmaz et al. found that specific bacterial components in the human intestinal flora could activate natural defense mechanisms, such as inducing the host to produce high levels of circulating natural antibodies that can recognize and kill *Plasmodium*, thereby efficiently protecting the host and preventing malaria spread (Yilmaz et al., 2014). Therefore, gut microbiota modulation probably has outstanding potential as a novel approach to prevent or improve severe malaria (Waide and Schmidt, 2020). Additionally, Villarino et al. found that the severity of malaria in mice was profoundly affected by the composition of the gut microbiota, in which, susceptible mice that accepted laboratory-cultured yogurt made from the species *Lactobacillus* and *Bifidobacterium* displayed a decreased parasite burden (Villarino et al., 2016). Previous studies showed that the MIF level could be changed by probiotic treatment, indicating an interaction between intestinal microbiota and MIF. Importantly, commonalities of intestinal microbial communities between African children and *Plasmodium*-susceptible mice suggest that the possibility of probiotics modulating the mouse gut microbiota to control severe malaria may also be suitable for humans (Villarino et al., 2016). Therefore, by using C57BL/6 and PbA to build a murine CM (MCM) model that can mimic human CM (HCM), exploring the intestinal microbial changes caused by *Plasmodium* infection and the factors that regulate this change (such as MIF) can help to identify biomarkers associated with severe outcomes of CM.

In brief, the study focused on the regulation of MIF on the host gut microbiota in CM models based on WT and MIF knockout C57BL/6 mice infected with PbA to reveal a potential mechanism that MIF regulates CM. The results may bear the expectation that a synergistic treatment of MIF and intestinal flora can more effectively improve the severity of malaria in malaria treatment targeting MIF in the future.

## Materials and methods

### Mice

Eight- to 10-week-old male C57BL/6 mice (22–30 g weight) were used in the present study. C57BL/6 (wild type, WT) mice were purchased from HNSJA Co., Ltd., Changsha, China. *Mif*^−^/^−^ mice with a C57BL/6 genetic background were constructed with the assistance of a professional institute (Gempharmatech Co., Ltd., Jiangsu, China). All animals were maintained in appropriate living conditions (25°C ± 3°C) and were entitled to freely consume a standard diet and pure water daily. Protocols for the use of animals were approved by the Institutional Animal Care and Use Committee of the Hubei University of Medicine (HBMU-S20160414).

### Parasite and establishment of murine cerebral malaria

*Plasmodium berghei* ANKA (PbA) was kindly provided by Professor Wenyue Xu (Third Military Medical University, China) and stored in liquid nitrogen before usage. The parasites were revived by intraperitoneal inoculation in WT C57BL/6 mice, which did not act as donor mice until at least the third passage.

Before the sequencing of intestinal flora, the infection and model of murine cerebral malaria (MCM) were first established and confirmed. In brief, WT (*n* = 10) and MIF KO (*n* = 10) mice were intraperitoneally inoculated with approximately 1 × 10^6^ infected red blood cells (iRBCs) from the donor mice in sterile saline (0.9% NaCl) with a volume of 200 μl. From the first day post inoculation (dpi), parasitemia was determined using thin blood smears made from mouse tails and stained with Giemsa. For each mouse, infected red blood cells (iRBCs) in at least 1,000 RBCs were counted under light microscopy. Moreover, assessment of blood–brain barrier permeability was performed as previously reported with some modifications (Taniguchi et al., 2015; Huang et al., 2019). In brief, five mice were randomly taken from both the WT + PbA and KO + PbA groups at 6 dpi and two uninfected mice were injected intravenously with 200 μl of 1% Evans blue dye (Sigma-Aldrich, St Louis, United States) and allowed to circulate in the body for 30 min. Then the mice were anesthetized and sent for heart perfusion with 20 ml of sterile saline (0.9% NaCl). After image documentation, whole brain tissues were placed in formamide for 48 h at 37°C to extract Evans blue. Then, aliquots (100 μl) of the solution of each mouse were analyzed on a microtiter plate for three repeats. Absorbance was measured at 630 nm on an ELISA reader and compared to a preformed standard curve following the document.

After the approach of MCM establishment was confirmed, mice that were randomly assigned to the WT + PbA (WPbA, *n* = 12) and KO + PbA (KPbA, *n* = 12) groups were infected with PbA under the same conditions for sequencing of intestinal flora. For the WT control (WT, *n* = 8) and KO control (KO, *n* = 7) groups, mice were only injected with sterile saline (0.9% NaCl) in the same volume.

### Preparation of gut contents

Gut content samples were collected at 6 dpi, when manifestations of MCM have appeared in most mice, but they had not yet widely died. Only the surviving mice were sacrificed to extrude feces from the whole gut into a sterile EP tube. After being immediately frozen in liquid nitrogen, all gut content samples were transferred to a professional sequencing company (Novogene Bioinformatics Technology Co., Ltd. in Tianjin, China) with dry ice.

### DNA extraction, PCR amplification, library construction, and sequencing

Genomic DNA of the fecal samples was extracted using the CTAB or SDS method. After detecting the purity and concentration of DNA by agarose gel electrophoresis, an appropriate amount of sample DNA was diluted to 1 ng/μl with sterile water to be the PCR template. Then, specific primers based on 16S rRNA (515F and 806R, 392 bp), Phusion® High-Fidelity PCR Master Mix with GC Buffer and high-efficiency high-fidelity enzyme from New England Biolabs were used for PCR to ensure the efficiency and accuracy of amplification. PCR products were verified by 2% agarose gel electrophoresis, and only the qualified PCR products were purified by magnetic beads and quantified.

The mixed PCR products in equal amounts were finally purified with the Qiagen Gel Extraction Kit (Qiagen, Germany). Sequencing libraries were constructed with a TruSeq® DNA PCR-Free Sample Preparation Kit (Illumina, United States) and quantified by Qubit and Q-PCR. After the library was qualified, NovaSeq6000 was used for on-machine sequencing.

### Sequencing data processing and statistical analysis

According to the barcode sequence and PCR primers, the data of each sample were split from the offline data. After truncating the barcode and primer sequences, FLASH was used to splice the reads of each sample to obtain the original tag data (raw tags; Magoč and Salzberg, 2011). The raw tags were subjected to strict filtering (Bokulich et al., 2013) to obtain high-quality tag data (clean tags). Referring to the tag quality control process of QIIME (Caporaso et al., 2010), the chimera sequences were detected by comparison with the species annotation database (Rognes et al., 2016) and removed (Haas et al., 2011) to obtain the final effective data (effective tags).

The Uparse algorithm was used to cluster all the effective tags of all samples (Edgar, 2013). By default, the sequences were clustered into operational taxonomic units (OTUs) based on 97% identity and the sequences with the highest frequency in the OTUs were screened out as representative sequences. Species annotation analysis was performed using the Mothur method and the SSUrRNA database (Quast et al., 2013) of SILVA138 (with a threshold of 0.8 ~ 1; Wang et al., 2007). Meanwhile, the community composition of each sample was counted at all taxonomic levels: kingdom, phylum, class, order, family, genus and species. A rapid multiple sequence alignment was performed using MUSCLE software to obtain the phylogenetic relationship of all representative sequences of OTUs (Edgar, 2004). Finally, the data of each sample were homogenized, and the sample with the least amount of data was used as the standard for homogenization. The subsequent alpha diversity analysis and beta diversity analysis were based on the homogenized data.

The observed_species, Chao1, Shannon and PD_whole_tree were all calculated in QIIME software (Version 1.9.1). The rarefaction curves and species accumulation curves were all plotted using R software (Version 2.15.3). Differences among groups regarding the alpha diversity index were also obtained in R software using Tukey’s test and the Wilcoxon test.

The UniFrac distance was calculated by QIIME software (Version 1.9.1), and the unweighted pair-group method with arithmetic mean (UPGMA) sample clustering tree was constructed. PCoA graphs were drawn using R software (Version 2.15.3). PCoA analyses were completed with the ade4, ggplot2 and vegan packages in R software. Differences among groups regarding the beta diversity index were also obtained in R software using the Tukey’s test and the Wilcoxon test.

LEfSe analysis was performed using LEfSe software, and the default setting of the LDA score was 4. A permutation test between groups under each classification level (phylum, class, order, family, genus, species) was performed with Metastats analysis by using R software to obtain the *p-*value, and then the *p*-value was corrected with the Benjamini and Hochberg False Discovery Rate method to acquire the *q*-value (White et al., 2009). Anosim and MRPP analyses were finished with the anosim and mrpp functions of the R packages, respectively. Species with significant differences between groups were also analyzed by *T*-test and plotted with R software.

## Results

### Confirmation of infection and murine cerebral malaria

**Figure 1:**
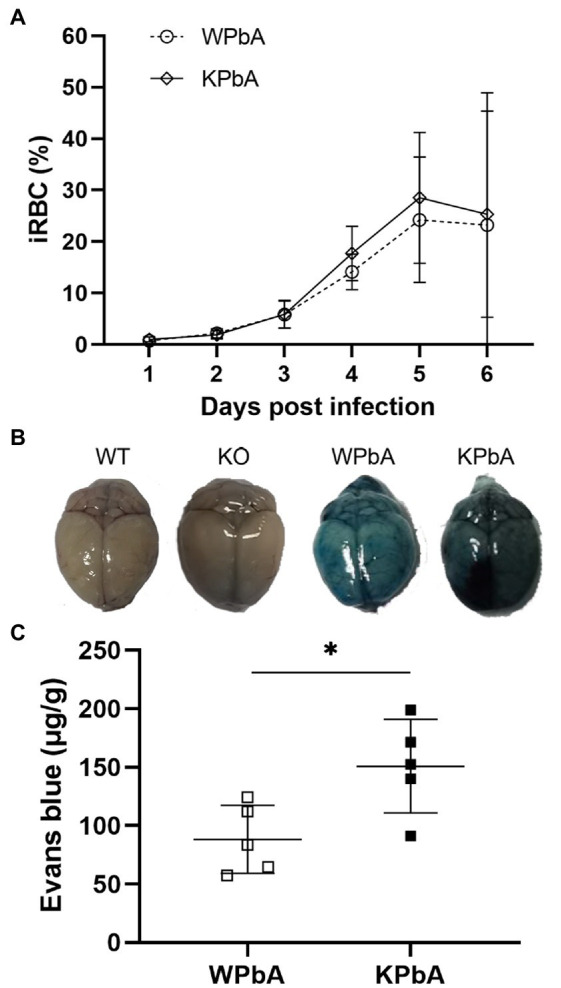
Differences in the pathology between WT and KO mice infected with PbA. **(A)** Parasitemia was monitored in both the WPbA and KPbA groups, daily. Parasitemia values represent the means ± SD from 10 mice in each group. **(B)** Mice were injected intravenously with Evans blue at 6 dpi. Uninfected mice were used as controls. **(C)** The amounts of EB per g of brain from infected mice are shown (*n* = 5). Statistical analysis was performed using the *T*-test. ^*^p < 0.05.

As shown in Figure 1A, an average of 23.23% and 25.33% iRBCs were detected in mice from the WPbA and KPbA groups at 6 dpi, respectively. Parasitemia was slightly higher in the KPbA group than in the WPbA group from 4 to 6 dpi. Importantly, the leakage of Evans blue dye into the brains indicated that the collapse of the blood–brain barrier and the development of MCM in both PbA-infected groups occurred at 6 dpi (Figure 1B). Significantly more severe damage to blood–brain barrier permeability appeared in the KPbA mice (Figure 1C). Of course, the closely related clinical symptoms of MCM (Carroll et al., 2010) including ruffled fur, ataxic gait, hunching, limb paralysis, and even convulsions or coma, were also observed at 6 dpi. These results indicated that the infection was sustained and MCM has appeared in the WPbA and KPbA groups at 6 dpi. The absence of MIF seems to play an adverse role in MCM in the present study.

By using the same approach for MCM establishment as above, mice prepared for analysis of intestinal flora have also showed MCM signs (except for one and three deaths in WPbA and KPbA before sequencing, respectively) at 6 dpi, including piloerection, mild ataxia in gait and decreased exploratory behavior, strength and reflexes and self-preservation, which are usually followed by severe CM and death, probably at any time of the next several days according to previous experiences and documents (Engwerda et al., 2005; McQuillan et al., 2011; Schmutzhard et al., 2012).

### Overview and quality assessment of sequencing data

As shown in Table 1, a total of 2,948,760 raw sequences were generated from all samples after sequencing. Among them, the number of filtered effective tags was 2,219,911 (average effective% = 76.90%), ranging from 58,864 to 68,731 with an average of 63,426 ± 2,883. An average of 98.12 ± 0.13 in Q30 (from 97.79 to 98.36) guaranteed the accuracy of sequencing. Effective tags were clustered with ≥97% sequence identity as the operational taxonomic units (OTUs), and a total of 29,836 OTUs were finally obtained, with an average number of 852 ± 39 (from 752 to 917). The number of observed species was 28,131 in total, and the mean number of all samples was 804 ± 38 (from 699 to 871). Interestingly, the mean number of OTUs in the WPbA group was higher than that in the WT group. However, compared with the KO group, the average number of OTUs was decreased in the KPbA group. The quantity of shared and unique OTUs among groups or different samples within a group were displayed using Venn diagrams or flower figures, respectively (Supplementary Figures 1A,B). Briefly, both PbA-infected groups had a reduction in the amount of shared OTUs relative to their corresponding control groups, with a greater reduction in KPbA mice. The rarefaction curves showed that the species diversity in each sample approached saturation levels, indicating that the sequencing depth had been progressively justified (Supplementary Figure 2A). The species accumulation boxplot tended to be flattened, indicating that the number of samples for sequencing was sufficient (Supplementary Figure 2B).

**Table 1:** Operational taxonomic unit (OTU)-based diversity indexes of gut microbiota during PbA infection in mice.

Sample name	Group	Raw PE (#)	Effective tags (#)	Base (nt)	Q30	GC%	Effective%	OTUs _ num	Observed _species	Chao1	Shannon	PD_whole _tree
WT1	WT	83,393	61,040	15,454,218	98.24	54.34	73.38	839	785	822.54	6.86	42.89
WT2		86,731	66,777	16,904,606	98.29	54.11	77.18	802	757	799.13	6.84	38.33
WT3		84,850	67,546	17,114,966	98.26	54.11	79.86	902	852	893.03	7.45	42.71
WT4		86,704	66,544	16,835,539	98.15	53.72	76.85	842	789	830.03	6.58	41.17
WT5		82,164	62,491	15,817,775	97.96	54.08	76.23	846	794	841.35	6.75	38.07
WT6		87,044	63,907	16,229,135	98.30	54.01	73.82	904	841	876.01	7.21	38.62
WT7		78,348	61,327	15,510,186	98.22	53.79	78.38	873	831	873.30	7.22	42.30
WT8		89,080	65,281	16,556,419	98.32	54.05	73.56	824	777	824.67	7.27	46.17
WPbA1	WPbA	76,120	62,080	15,777,822	98.24	53.99	82.06	853	790	828.61	7.32	40.13
WPbA2		84,771	66,746	17,008,817	98.34	54.39	79.39	896	844	895.35	7.10	38.83
WPbA3		82,672	63,199	16,055,022	98.36	54.32	76.88	876	836	888.32	7.30	38.68
WPbA4		79,189	60,828	15,552,203	98.09	54.23	77.74	878	819	844.21	7.44	38.20
WPbA5		80,517	59,890	15,287,290	98.05	54.05	75.17	840	836	887.51	7.20	41.33
WPbA6		83,183	59,644	15,256,057	98.09	54.13	72.60	917	871	899.24	7.75	42.25
WPbA7		83,765	62,076	15,803,548	98.04	53.8	74.62	863	818	849.70	7.48	41.38
WPbA8		77,164	62,203	15,872,823	97.79	54.3	81.41	845	794	835.91	7.31	36.97
WPbA9		80,663	63,667	16,345,325	98.08	53.95	80.21	914	855	901.13	7.64	39.15
WPbA10		90,875	65,626	16,703,798	98.12	53.64	72.77	908	855	894.00	7.46	41.94
WPbA11		94,143	68,731	17,612,809	98.12	53.93	74.02	842	790	840.55	7.34	40.67
KO1	KO	83,693	65,390	16,850,799	98.05	54.33	79.68	881	837	873.55	7.24	38.26
KO2		86,099	64,968	17,128,816	97.98	53.95	78.70	884	834	865.09	7.24	38.32
KO3		86,268	67,328	17,223,574	97.90	54.23	79.00	861	811	848.54	7.00	37.75
KO4		83,451	63,608	16,642,542	98.12	54.56	78.94	868	825	856.66	7.32	37.76
KO5		83,967	59,834	15,715,165	98.08	54.37	74.08	845	793	827.04	7.24	36.64
KO6		87,724	66,811	17,473,320	98.17	54.26	78.84	806	752	826.27	7.15	35.71
KO7		80,770	59,066	15,521,580	98.13	54.05	76.05	877	829	853.24	7.41	37.33
KPbA1	KPbA	80,365	59,578	15,313,589	98.10	54.38	75.42	818	775	809.58	6.94	36.85
KPbA2		87,741	66,140	17,175,530	97.83	54.39	77.49	824	758	794.32	7.04	40.41
KPbA3		83,509	59,473	16,073,771	98.12	54.16	76.19	865	813	861.73	7.25	37.48
KPbA4		83,315	64,557	16,696,819	98.15	54.13	79.35	865	811	860.10	7.19	37.76
KPbA5		82,798	62,824	16,347,210	98.12	54.55	78.16	791	746	780.52	7.04	39.19
KPbA6		83,441	58,864	15,731,794	98.10	54.54	74.63	795	755	808.28	6.97	35.45
KPbA7		82,352	61,630	16,819,705	98.15	54.18	80.82	805	765	817.25	7.16	34.48
KPbA8		92,947	67,682	17,565,926	98.07	54.33	74.79	752	699	750.89	6.89	33.04
KPbA9		88,944	62,555	16,495,818	98.24	54.75	73.40	835	794	844.93	7.01	36.51
Total		2,948,760	2,219,911	572,474,316	3,434	1,896	2,692	29,836	28,131	29,603	251.59	1362.72
Max		94,143	68,731	17,612,809	98.36	54.75	82.06	917	871	901.13	7.75	46.17
Min		76,120	58,864	15,256,057	97.79	53.64	72.60	752	699	750.89	6.58	33.04
Average		84,250	63,426	16,356,409	98.12	54.17	76.90	852	804	845.79	7.19	38.93
SD		4,072	2,883	710,104	0.13	0.25	2.66	39	38	35.97	0.25	2.68

WT and KO represent Control Groups (uninfected); WPbA and KPbA represent *Plasmodium berghei* ANKA-infected groups; Max, Mini, and SD represent the maximum value, minimum value, and standard deviation of the mean number, respectively.

### Overview of the relative abundance and clustering of species

**Figure 2:**
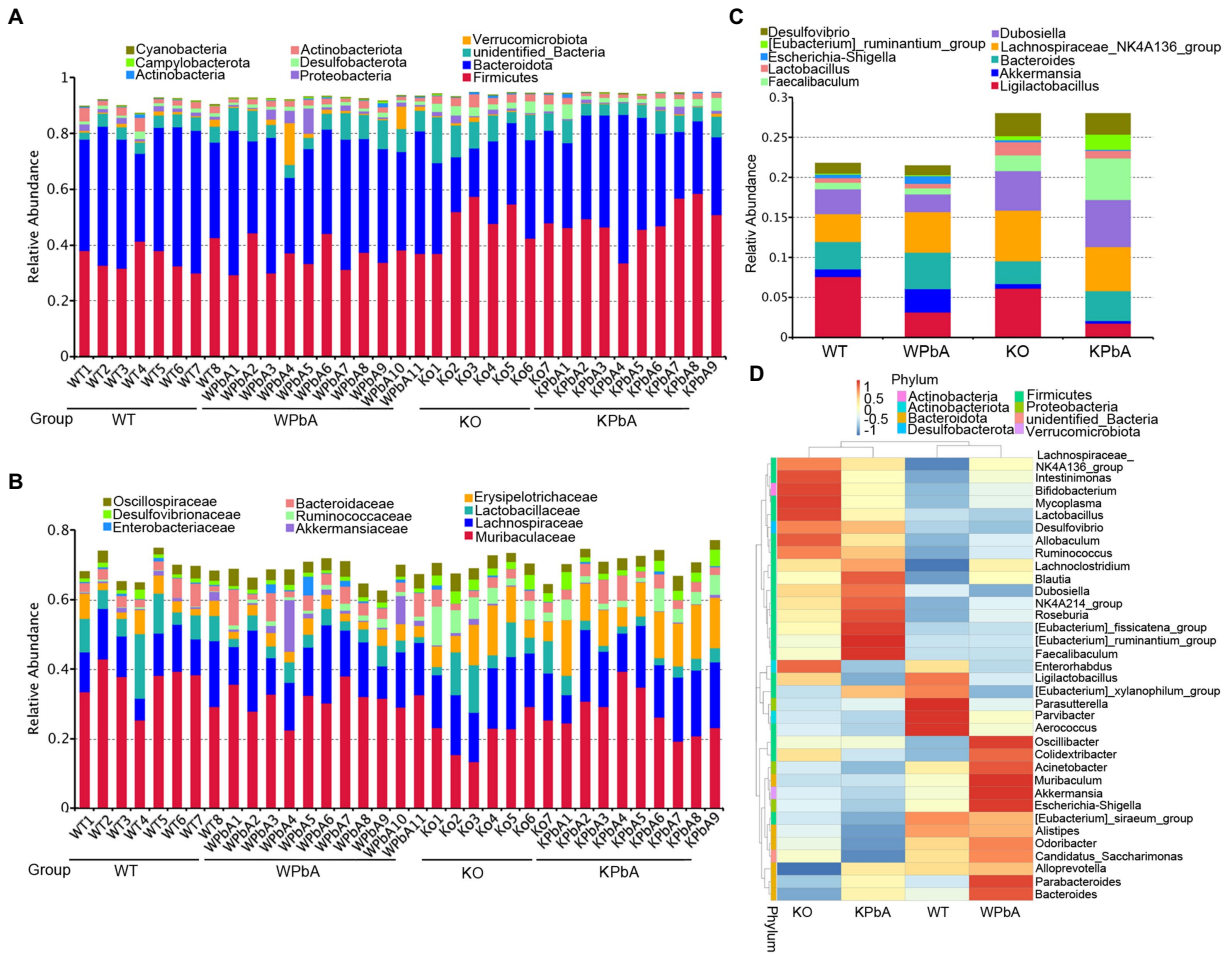
Variation in microbial community components at different taxonomic levels. **(A)** Proportions of bacterial communities at the phylum level. **(B)** Proportions of bacterial communities at the family level. **(C)** Proportions of bacterial communities at the genus level. Each column represents the constitution and proportion of the top 10 most abundant microbial communities in each individual or group. **(D)** Taxonomic heatmap of the top 35 most abundant microbial communities at the genus level. The left pattern represents the clustering trees at the genus level. The above profile displays clustering trees among different groups.

According to the annotation results, the top 10 phyla, families and genera with the largest abundance in each sample or group were used to generate the column accumulation charts of relative abundance. As shown in Figure 2A, the most abundant phylum was Firmicutes, with an average proportion of 41.71%, followed by Bacteroidota (37.19%). However, for different groups, the proportion of dominant phyla was different. Compared with the WT (35.85%) group, Firmicutes had no significant change in WPbA (36.04%) mice but was obviously increased in KO (48.68%) and KPbA (48.43%) mice. Bacteroidota was slightly decreased in WPbA (40.53%) compared to the WT (43.37%) group and was apparently decreased in the KO (28.09%) and KPbA (34.69%) groups. The remaining seven of the top 10 phyla (excluding unidentified bacteria, 6.78%) were all changed among the four groups and characterized by limited relative abundance, even less than an average proportion of 2.5%.

As shown in Figure 2B, the most abundant family was Muribaculaceae, with an average proportion of 29.59%, followed by Lachnospiraceae (14.89%), Erysipelotrichaceae (6.75%), and Lactobacillaceae (5.52%). Muribaculaceae and Lactobacillaceae were obviously reduced in the KO (21.92%, 7.97%) and KPbA groups (27.74%, 3.04%) compared with the WT (35.75%, 8.46%) and WPbA (31.51%, 3.84%) groups. In contrast, Lachnospiraceae and Erysipelotrichaceae were overall increased in the KO (16.27%, 8.22%) and KPbA groups (16.06%, 11.98%) compared with the WT (12.37%, 4.10%) and WPbA (14.88%, 3.45%) groups.

As shown in Figure 2C, the most abundant genus was *Lachnospiraceae_NK4A136_group*, with an average proportion of 5.06%, followed by *Ligilactobacillus* (4.43%), *Dubosiella* (3.91%) and *Bacteroides* (3.75%). *Ligilactobacillus* and *Bacteroides* were obviously reduced in the KO (6.18%, 2.82%) and KPbA groups (1.79%, 3.77%) compared with the WT (7.59%, 3.42%) and WPbA (3.19%, 4.56%) groups, respectively. Conversely, *Lachnospiraceae_NK4A136_group*, *Dubosiella* and *Faecalibaculum* were increased overall in the KO (6.36%, 4.89%, and 2.01%) and KPbA groups (5.52%, 5.88%, and 5.17%) compared with the WT (3.44%, 3.15%, and 0.80%) and WPbA (5.05%, 2.21%, and 0.81%) groups, respectively. It is interesting that *Akkermansia* was increased by approximately threefold in the WPbA group compared to the WT group, but decreased by twofold in the KPbA group compared to the KO group. Additionally, the top 35 genera in abundance were clustered at both the species and sample levels, and the heatmap of the results was shown in Figure 2D. Overall, mice in the WT and WPbA groups tended to cluster together, and the same applied to the samples in the KO and KPbA groups. Compared to the WT group, higher amounts of *Oscillibacter*, *Muribaculum*, *Akkermansia*, *Escherichia-Shigella*, *Parabacteroides* and *Bacteroides*, and decreased amounts of *Dubosiella*, *Desulfovibrio* and *Lactobacillus* were enriched in the WPbA group. However, for the KO group, the most abundant species clustered were *Intestinimonas*, *Bifidobacterium*, *Mycoplasma*, and *Lactobacillus*. Compared to the KO group, the KPbA group was characterized by higher amounts of *[Eubacterium]_fissicatena_group*, *[Eubacterium] _ ruminantium_group*, *Faecalibaculum* and *Roseburia*, and decreased amounts of *[Eubacterium]_siraeum_group*, *Alistipes*, *Odoribacter* and *Candidatus_Saccharimonas*.

### Diversity of bacterial community within groups

**Figure 3:**
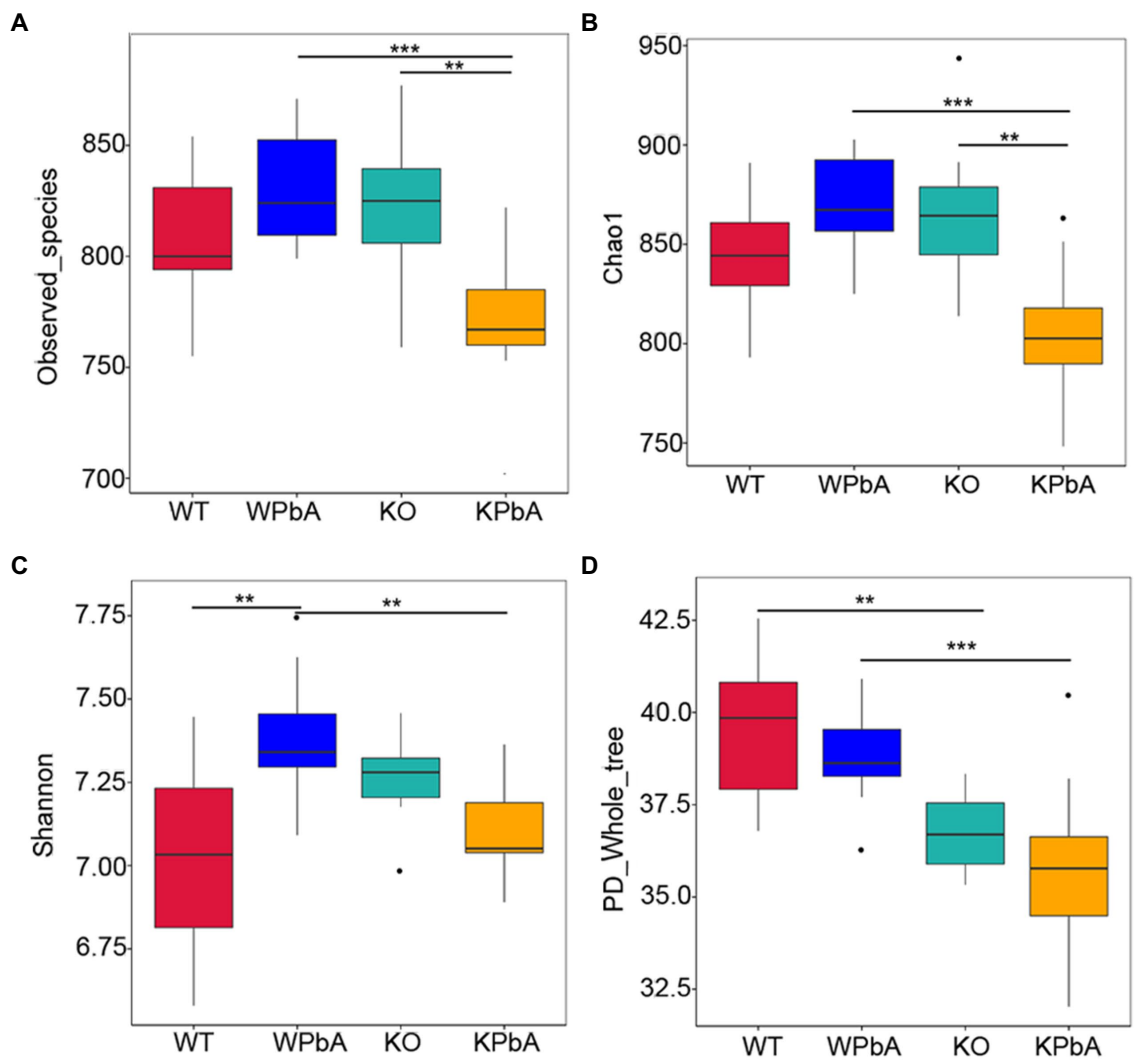
The richness and diversity of microbes within communities by alpha analysis. **(A)** Observed_species, **(B)** Chao1, **(C)** Shannon, and **(D)** PD_whole_tree. Differences among groups regarding the alpha diversity index were obtained in R software using the Wilcoxon test. Significant differences are indicated *via* ^**^p < 0.01 and ^***^p < 0.001.

The richness and diversity of microbes within communities were assessed by alpha diversity analysis, including observed_species, Chao1, Shannon and PD_whole_tree (Table 1; Figure 3). Compared with the WT group, the average number of OTUs and the median of observed_species, Chao1 and Shannon in the WPbA group were all increased. In contrast, compared with the KO group, the above indicators in the KPbA group were all decreased. Slightly different, for PD_whole_tree, a gradual downward trend in the four groups WT, WPbA, KO, and KPbA was observed.

Observed_species (*p* = 0.0074, *p* = 0.0006, respectively; Wilcox rank test, respectively) and Chao1 (*p* = 0.0018, *p* = 0.0001; Wilcox rank sum test, respectively) were all significantly decreased in the KPbA group when compared with the WPbA and KO groups. However, the median values of these two indexes were all increased in the WPbA and KO groups compared with the WT group, but there was no significant difference (Figures 3A,B). The Shannon value was significantly elevated in the WPbA group (*p* = 0.0017; Wilcoxon rank sum test) compared with the WT group, but conversely, it was significantly decreased in the KPbA group (*p* = 0.0032; Wilcoxon rank-sum test) compared with the WPbA group (Figure 3C). In contrast, PD_whole_tree was significantly decreased in the KO group (*p* = 0.0024; Wilcoxon rank-sum test) compared to the WT group and significantly reduced in the KPbA group (*p* = 0.0009; Wilcoxon rank-sum test) compared with the WPbA group (Figure 3D).

### Comparative analysis of the bacterial communities among groups

**Figure 4:**
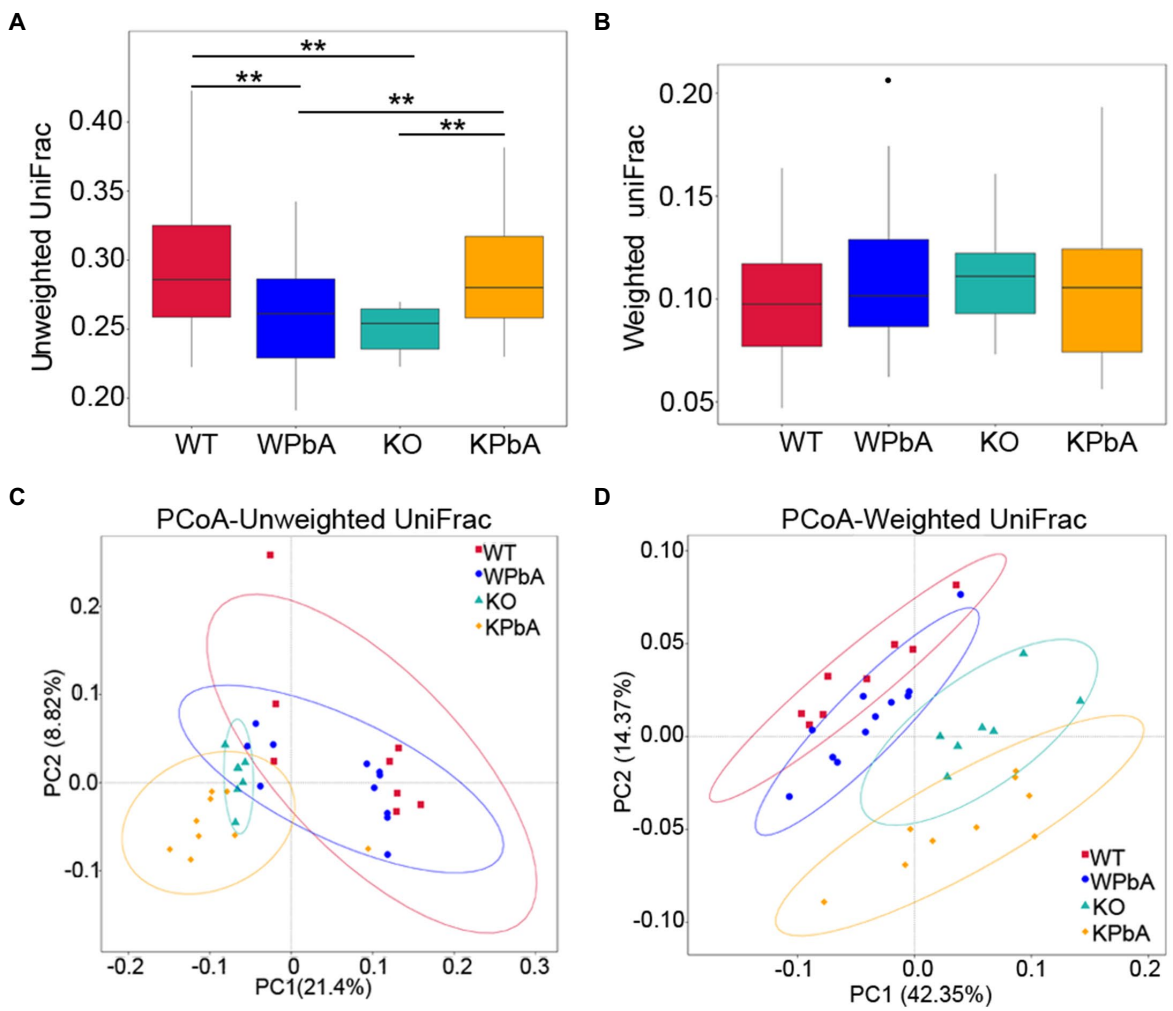
Comparative analysis of the microbial community composition among different groups by beta diversity. **(A)** Unweighted UniFrac, **(B)** weighted UniFrac, **(C)** principal coordinates analysis (PCoA) based on unweighted, and **(D)** weighted UniFrac distance matrixes for intestinal contents from four groups involved. Significant differences are indicated *via* ^**^p < 0.01.

Beta diversity was used for comparative analysis of the microbial community composition among different groups. Unweighted UniFrac analysis showed that medians were significantly lower in both WPbA (*p* = 0.0041; Wilcox rank-sum test) and KO (*p* = 0.0004; Wilcox rank-sum test) mice than in WT mice. However, it was significantly higher in the KPbA mice (*p* = 0.0001, *p* = 0.0008, respectively; Wilcoxon rank sum test) than in the KO and WPbA groups (Figure 4A). In contrast, the median values among the four groups showed no significant difference by weighted UniFrac analysis (Figure 4B).

To assess the structural similarity of species composition, PCoA based on unweighted UniFrac and weighted UniFrac was performed on each sample (Figures 4C,D). The results presented a relatively consistent clustering trend by the two methods. Anosim and MRPP are two similar analytical methods that can be used for significance testing of the differences in microbial community structure between groups. The results showed that differences among all groups were significant and greater than those within group samples (Table 2), indicating that both MIF gene knockout and PbA infection could cause significant changes in the gut microbial community. Additionally, the 35 samples were subjected to UPGMA cluster analysis with a weighted UniFrac distance matrix (Supplementary Figure 3). The results revealed that the WT and WPbA groups or the KO and KPbA groups were clustered into two separate branches at the phylum level.

**Table 2:** Within- vs. among-group dissimilarities analysis *via* analysis of similarities (ANOSIM) and multi response permutation procedure (MRPP).

Method	ANOSIM^a^		MRPP^b^			
Group	*R*-value	*P*-value	A	Observed-delta	Expected-delta	Significance
WPbA-WT	0.549	0.001	0.094	0.307	0.339	0.001
KO-WT	0.999	0.001	0.213	0.313	0.398	0.002
KPbA-WPbA	0.988	0.001	0.224	0.307	0.396	0.001
KPbA-KO	0.575	0.001	0.084	0.313	0.341	0.001

a The R-value is between −1 and 1, and an R-value greater than 0 indicates that differences between groups are greater than those within group. Significance < 0.05 represents a significant difference.

b A value greater than 0 indicates that differences between groups are greater than those within group. Significance < 0.05 represents a significant difference. A smaller observed-delta value indicates a lower difference within a group; a larger expected-delta value indicates a greater difference between the groups.

### Potential biomarker discovery

**Figure 5:**
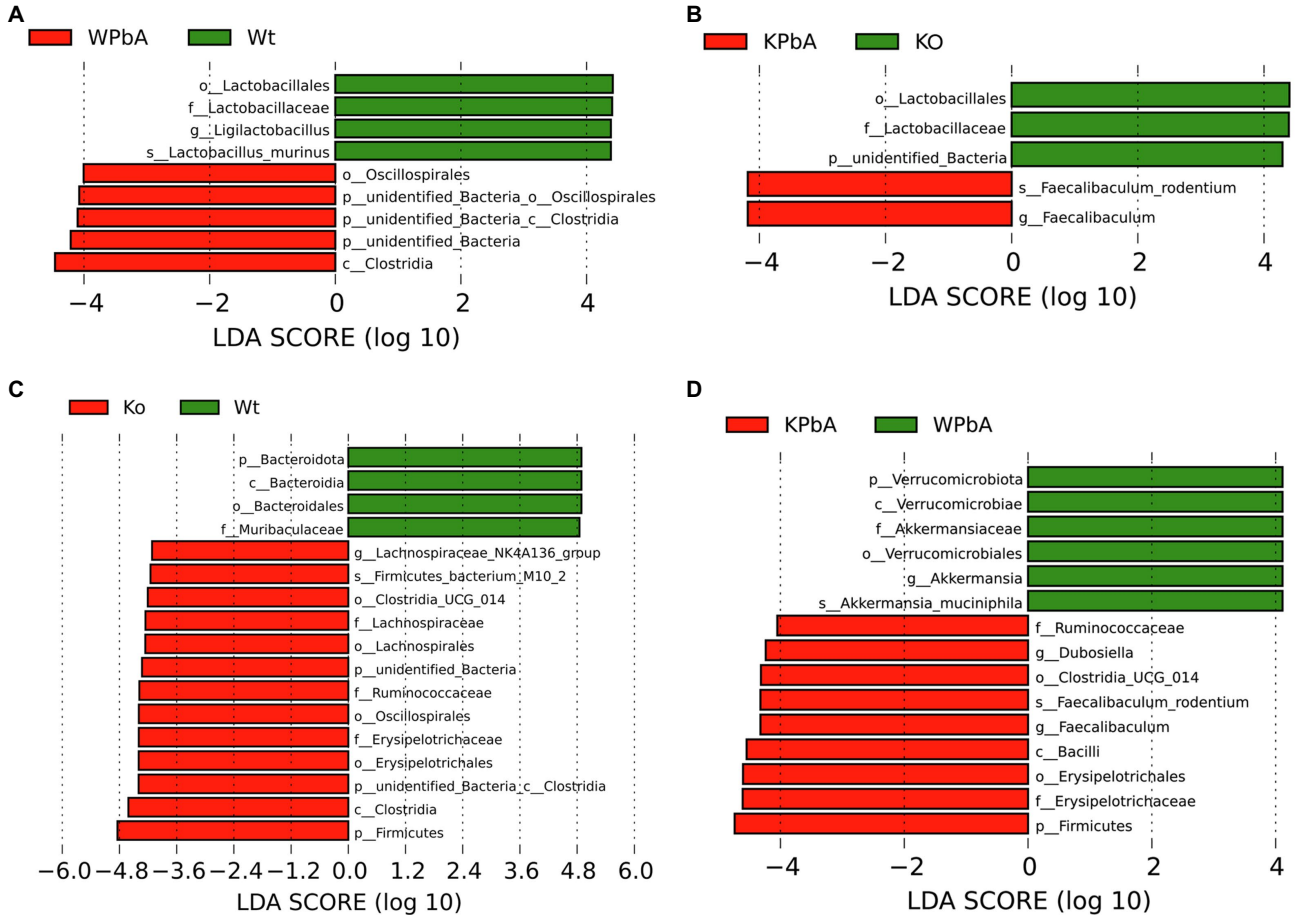
The differentially abundant bacterial taxa identified by LEfSe among different groups. **(A)** Linear discriminant analysis (LDA) score distribution between the WT and WPbA groups. **(B)** LDA score distribution between the KO and KPbA groups. **(C)** LDA score distribution between the WT and KO groups. **(D)** LDA score distribution between the WPbA and KPbA groups. The histogram of LDA value distribution only showed the species whose LDA score was greater than the default setting (threshold > 4), which represented the biomarkers with significant differences between groups.

The LDA effect size (LEfSe) analysis was able to find statistically significant biomarkers between groups. As shown in Figure 5, potential biomarkers existed at different taxonomic levels. At the genus and species level, the biomarker of WT mice was *Ligilactobacillus* (*Lactobacillus murinus*) compared with WPbA but was *Faecalibaculum* (*Faecalibaculum rodentium*) for the KPbA compared with the KO groups (Figures 5A,B). Compared to WT mice, *Lachnospiraceae_NK4A136_group* (genus) and *Firmicutes_bacterium_M10-2* (species) were highlighted as significantly different biomarkers in the KO group (Figure 5C). Interestingly, when comparing WPbA with KPbA, the biomarkers for WPbA were *Akkermansia* (*Akkermansia_muciniphila*), and the biomarkers for KPbA included *Dubosiella* and *Faecalibaculum* (*Faecalibaculum rodentium*; Figure 5D).

## Discussion

The elevated level of MIF, an important immune molecule in humans, was reported to be a possible risk factor for death in patients with CM (Jain et al., 2009), making it a potential molecular target for improving severe malaria. In contrast, another study revealed that MIF had a protective role in CM (Awandare et al., 2007), implying that MIF probably has both protective and adverse effects against malaria. In general, its actions and mechanisms seem very complicated and remain unclear. Altering the microbial community of patients may be one of the pathways by which MIF affects CM. The results of gut microbiota sequencing showed that the OTU clustering, as well as the alpha and beta diversity in the four groups of the present study all had obvious changes.

For the top two dominant phyla, Firmicutes was elevated by ~12% in both KO and KPbA mice, but Bacteroidota was decreased by 15.28% in KO and 5.8% in KPbA compared to the WT and WPbA groups, respectively. A previous study reported that immune protection in the intestine depends on the development of Bacteroidetes (Sequeira et al., 2020). Therefore, its reduction in the KO and KPbA groups may hint that the deficiency of MIF would weaken, at least partially, microbial immune protection in the intestine of the PbA-infected and uninfected hosts. Additionally, the Firmicutes/Bacteroidetes (F/B) ratios in KO and KPbA mice were approximately 2.1 and 1.6 times as much as those in the WT and WPbA groups, respectively. Studies have shown that F/B ratios were significantly higher in prostate enlargement patients (Takezawa et al., 2021), spontaneously hypertensive rats (Yang et al., 2015), autistic individuals and spinal-onset amyotrophic lateral sclerosis patients (Strati et al., 2017). These results seemed to suggest that the increase in F/B was probably a potential risk marker for gut dysbiosis in MIF-deficient mice. Muribaculaceae, as the dominant family in the Bacteroidota phylum, had a similar change trend. Muribaculaceae was decreased by 13.83% in the KO and 3.77% in the KPbA groups compared to the WT and WPbA groups, respectively. Studies have shown that members of Muribaculaceae represent major foragers of mucin monosaccharide (Pereira et al., 2020), which forms a natural barrier in the human intestine to avoid intimate contact between virulent microbes and the intestinal wall. Therefore, MIF deficiency appears to affect uninfected and PbA-infected individuals by reducing the abundance of intestinal mucin-degrading bacteria. Conversely, Erysipelotrichaceae was increased by 4.12% in the KO and 8.53% in the KPbA groups compared to the WT and WPbA groups, respectively. Members of this family appear to be highly immunogenic, play an important role in gastrointestinal inflammation-related diseases, and cause host lipid-related metabolic disturbances (Kaakoush, 2015). Characterizing important changes in this family in KPbA mice may ultimately provide promising microbial targets to combat the metabolic disorders caused by malaria. As one of the most common probiotic families (Zawistowska-Rojek et al., 2022), Lactobacillaceae was significantly lower in the WPbA and KPbA groups than in their corresponding control groups, but was less affected by MIF knockout, indicating that it is mainly PbA infection that caused the decrease in Lactobacillaceae.

The LEfSe analysis in this study identified a number of biomarkers with significant differences between groups at different taxonomic levels. Here, we focus on the species level. When compared with WT, the relative abundance of *Lactobacillus murinus* (belonging to the genus *Ligilactobacillus*), one of the natural commensal gut bacteria in the intestine of healthy individuals (Lebovitz and Theus, 2019), was dramatically decreased in WPbA. Several studies have revealed the positive functions of *L. murinus in* antimicrobial production (Nardi et al., 2005), protection of the intestinal barrier (Delucchi et al., 2017) and neonatal necrotizing enterocolitis (Isani et al., 2018), indicating that *L. murinus* was probably also a promising probiotic candidate for CM improvement. When compared with the KO group, the biomarker was *Faecalibaculum* (*Faecalibaculum rodentium*) for the KPbA group. Similarly, when compared with WPbA, the biomarkers for KPbA included significantly increased *Faecalibaculum* (*Faecalibaculum rodentium*) and *Dubosiell* and decreased *Akkermansia* (*Akkermansia_muciniphila*). Interestingly, these three genera/species microbiota were all beneficial for the production of short-chain fatty acids (SCFAs; Shen et al., 2021; Ye et al., 2021), which, as one of the safeguards for maintaining gut health, plays an important role in strengthening epithelial barrier integrity and inhibiting inflammation. However, a previous study found that oral administration of *F. rodentium* in antibiotic-treated resilient *Ephx2* KO mice led to depression-like behaviors (Wang et al., 2021). Another study reported that *Dubosiella* was positively correlated with glycolipid metabolism disorders (Miao et al., 2022). *Akkermansia* spp., as important mucus-degrading bacteria, have significant anti-inflammatory activity and are considered as promising probiotics (Romano et al., 2021; Ye et al., 2021). Meanwhile, significant increases in *Lachnospiraceae_NK4A136_group* (genus) and *Firmicutes_bacterium_M10-2* (species) were also highlighted in the KO group compared with the WT group. The *Lachnospiraceae_NK4A136_group* represents a gut bacterium naturally inhabiting healthy individuals (Stadlbauer et al., 2020). Interestingly, it is also a producer of butyrate, which is an important SCFA (Gheorghe et al., 2022) and has a positive role in maintaining the gut barrier integrity of mice (Hu et al., 2019). However, the function of *Firmicutes_bacterium_M10-2* is still poorly understood in both humans and rodents. In brief, two interesting findings were discovered in the LEfSe analysis: 1) The probiotic *Ligilactobacillus* (*Lactobacillus murinus*) was significantly reduced in WPbA mice compared to the WT group, and a candidate probiotic *Akkermansia* (*Akkermansia_muciniphila*) was significantly reduced in the KPbA mice compared to the WPbA group. 2) MIF knockout mice, uninfected or infected with PbA, showed significant enrichment of producers of short-chain fatty acids; however, the predominant biomarkers were different between the two groups. The above results indicated that PbA infection could lead to an apparent decrease in the abundance of different probiotics or potential probiotics in WT and MIF KO mice. However, whether MIF deficiency is likely to play a beneficial role in malaria by upregulating the abundance of producers of short-chain fatty acids or otherwise exert adverse effects through other pathways, as previously reported or in an unknown way, still needs further exploration. Most likely, the role of different probiotics or potential probiotics changes in food habits of individuals as well as the pathogenesis of parasitic infection in human beings, which may culminate with short-chain fatty acids effects through different pathways.

In addition, Taniguchi et al. found that PbA infection resulted in significant changes in host intestinal pathology and microbiota (Taniguchi et al., 2015). Guan et al. found that the gut microbiota of C57BL/6 mice had been rebuilt on day 4 after PbA infection (Guan et al., 2021). However, different changes in the gut microbiome and significant biomarkers were reported in their studies. Studies have even found that mice from different commercial suppliers showed differences in gut bacterial communities and affected their susceptibility to *Plasmodium* (Ivanov et al., 2008, 2009). This may suggest that differences in gut microbiota are an important factor in whether different individuals develop severe malaria in the clinic. Therefore, the development of adjuvant therapy based on probiotic or harmful biomarkers screened in a single study will have great limitations. In other words, the bacterial flora changes and advantageous biomarkers reported in different studies may be different, but the consistent changes and shared significant biomarkers in different studies, in our opinion, are probably the key factors that affect the severity of different malaria patients and act as the most potential targets for improving severe malaria, including CM. By conducting comprehensive analysis on the limited documents about the influence of intestinal microbes on CM (Taniguchi et al., 2015; Guan et al., 2021) and biomarkers in the present study, it seems that the genus *Lactobacillus* that was frequently screened out probably be most responsible for CM.

This study revealed interesting changes in the gut microbiota of MIF KO and WT C57BL/6 mice before and after PbA infection. However, this study also had some limitations. Result in the present study showed an adverse role of MIF deficiency in mice with CM. Nevertheless, it is unknown how MIF affects the severity and pathogenesis of CM after changing the intestinal flora. A dynamic observation of the gut microbiota and the corresponding pathological, metabolic and immune changes is lacking. In future studies, we should also fully explore whether the addition of the identified potential probiotic biomarkers or the elimination of harmful bacteria with specific antibiotics can reduce the severity of CM and their microorganism-dependent mechanisms.

In summary, this study further proved the gut microbiota changes in C57BL/6 mice caused by PbA infection and found that MIF deletion directly affected the changes in the gut microbiota of C57BL/6 mice before and after PbA infection. This finding reveals a potential mechanism by which MIF regulates CM. The obtained potential biomarkers combined with MIF will also provide a promising idea to develop combined drugs for improving CM in the future.

## Data availability statement

The datasets presented in this study can be found in online repositories. The names of the repository/repositories and accession number(s) can be found at: https://www.ncbi.nlm.nih.gov/bioproject/PRJNA852657.

## Ethics statement

The animal study was reviewed and approved by Institutional Animal Care and Use Committee of the Hubei University of Medicine (HBMU-S20160414).

## Author contributions

WG, YZ, YX, and JL conceived the study, participated in its design, performed the data analysis and interpretation, and drafted the manuscript. WG, YZ, SY, KG, SC, XH, HS, and YX carried out the experiments. WG, YZ, YX, and JL participated in analyzing and interpreting the data. All authors contributed to the article and approved the submitted version.

## Funding

This work was supported by grants from the Post-Graduates of Hubei University of Medicine (2018QDJZR17 and 2016QDJZR04), foundation from the Research Project of Hubei Provincial Department of Education (Q20192102 and Q20172102), and partially supported by the Principle Investigator Program of Hubei University of Medicine (HBMUPI202101).

## Conflict of interest

The authors declare that the research was conducted in the absence of any commercial or financial relationships that could be construed as a potential conflict of interest.

## Publisher’s note

All claims expressed in this article are solely those of the authors and do not necessarily represent those of their affiliated organizations, or those of the publisher, the editors and the reviewers. Any product that may be evaluated in this article, or claim that may be made by its manufacturer, is not guaranteed or endorsed by the publisher.
