# Supplementary material for: Deficiency of migration inhibitory factor influences the gut microbiota of C57BL/6 mice infected with Plasmodium berghei ANKA
Source: Front Microbiol. 2022 Aug 12;13:978644. doi: 10.3389/fmicb.2022.978644 (PMC9412183; doi:10.3389/fmicb.2022.978644)
Supplement: Supplementary file 1 [file Data_Sheet_1.DOCX]

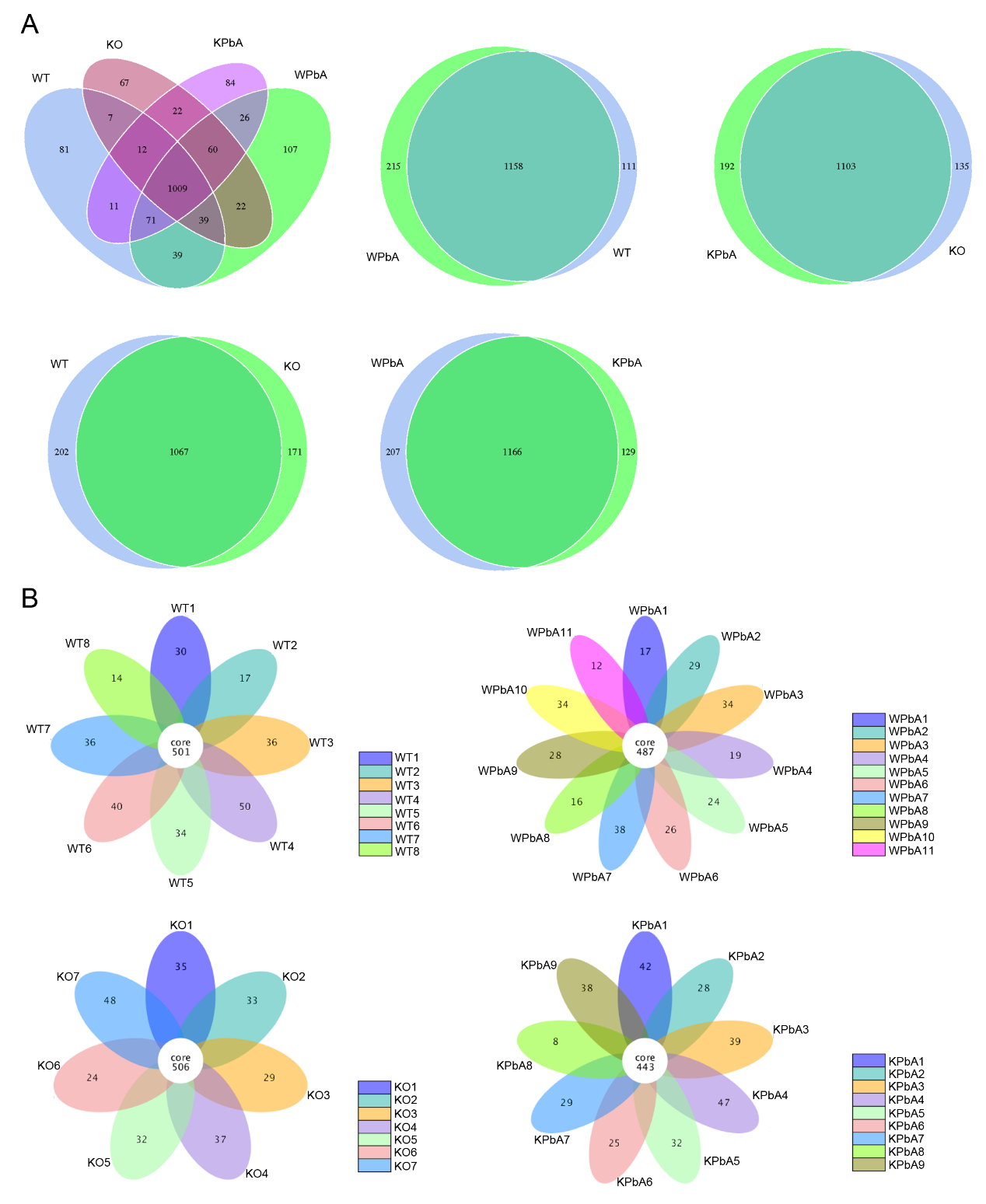


**Supplementary Figure 1 |** Quantity of shared and unique OTUs. **(A)** Venn diagrams represent the analysis among groups. **(B)** Flower diagrams displaying the analysis within groups.


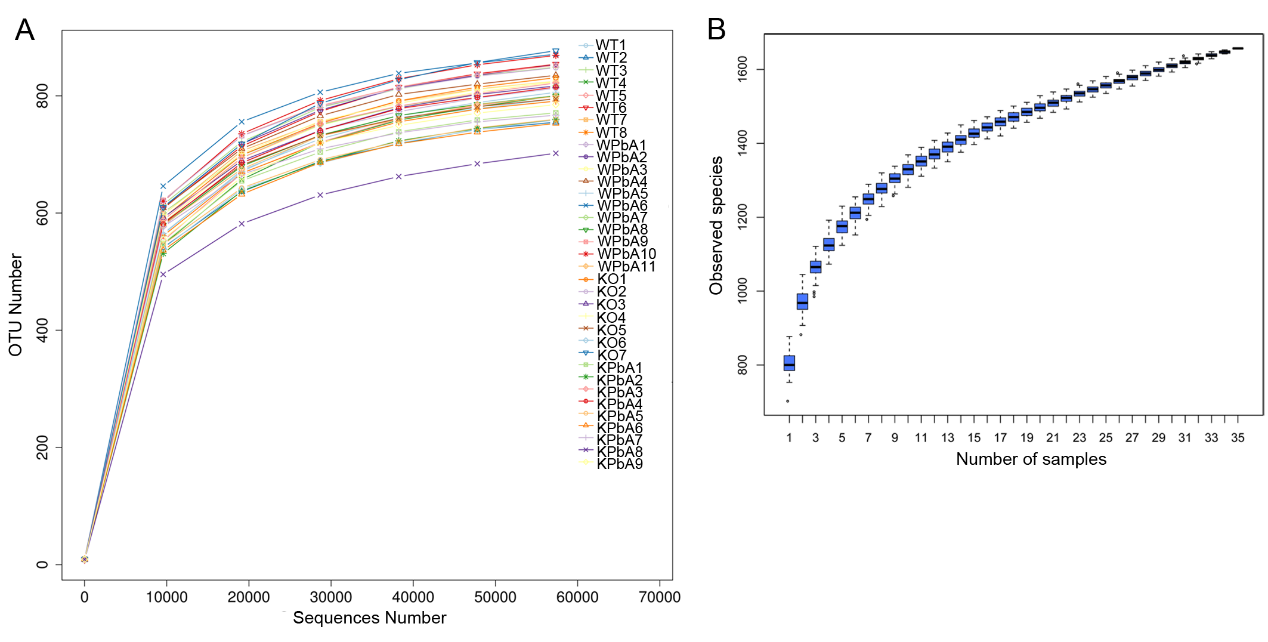


**Supplementary Figure 2 |** The assessment of sequencing depth and rationality of sample size. **(A)** Rarefaction curves for sequencing depth. **(B)** Species accumulation boxplot for rationality of the number of samples for sequencing.


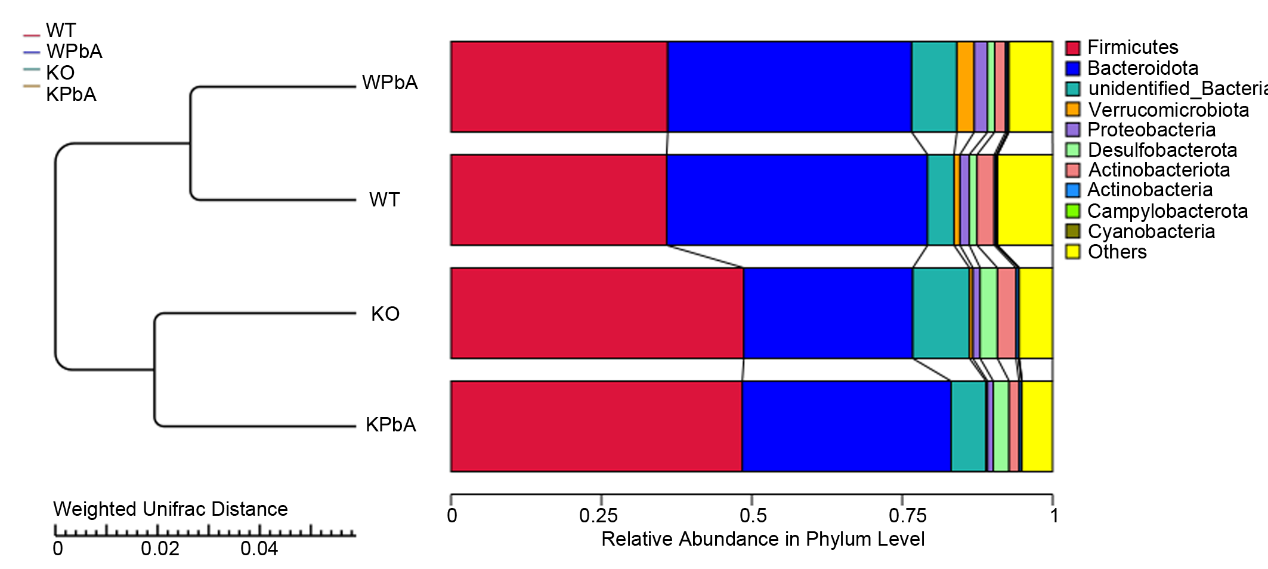


**Supplementary Figure 3 |** UPGMA clustering tree based on weighted UniFrac among different groups.
